# Exploratory LC-MS/MS-Based Proteomic and Lipidomic Profiling of Plasma Samples from Premature Coronary Artery Disease Patients: A Pilot Study in a South Asian Population

**DOI:** 10.3390/ijms27135684

**Published:** 2026-06-24

**Authors:** Iftikhar Ali Ch, Zahid Hasan, Zongkai Peng, Kamrul Islam, Amit Singh, Anayat Yousuf, Mohamed S. Aborahma, Ayan S. Zubair, Ali A. Rizvi, Nouraldeen Refai, Mohammad Omer Rana, Azhar A. Chaudhry, Fazal Jalil, Yasir Ali, Waseem Iqbal, Yusra Javed, Mishal Zehra, Tayyab Adeel Afzal, Ankur Kalra, Khurram Nasir, C Michael Gibson, Zhibo Yang, Nagib Ahsan

**Affiliations:** 1 South Oklahoma Heart Research, Oklahoma City, OK 73135, USA; 2 SSM Health Saint Anthony Hospital, Oklahoma City, OK 73102, USA; 3 Department of Chemistry and Biochemistry, University of Oklahoma, Norman, OK 73019, USA; 4 Department of Biochemistry and Physiology, University of Oklahoma Health Campus, Oklahoma City, OK 73104, USA; 5 Dodge Family College of Arts and Sciences, University of Oklahoma, Norman, OK 73019, USA; 6 Department of Biology, University of Central Oklahoma, Edmond, OK 73034, USA; 7 CPE Institute of Cardiology, Wazirabad 52000, Pakistan; 8 Armed Forces Institute of Cardiology, Rawalpindi 46000, Pakistan; 9 Department of Biotechnology, Abdul Wali Khan University, Mardan 23200, Pakistan; 10 Chughtai Lab, Rawalpindi 46000, Pakistan; 11 Department of Cardiology, Dow University of Health Sciences, Karachi 74200, Pakistan; 12 Oklahoma Medical Research Foundation, Oklahoma City, OK 73104, USA; 13 Division of Cardiology, Department of Medicine, State University of New York (SUNY), Upstate Medical University, Syracuse, NY 13210, USA; 14 Division of Cardiovascular Prevention and Wellness, Department of Cardiology, Houston Methodist DeBakey Heart and Vascular Center, Houston Methodist-Rice Digital Health Institute (HM-Rice DHI) Center, Houston, TX 77030, USA; 15 Baim Institute for Clinical Research and PERFUSE, Harvard School of Medicine, Boston, MA 02115, USA; 16 Mass Spectrometry, Proteomics and Metabolomics Core Facility, Stephenson Life Sciences Research Center, University of Oklahoma, Norman, OK 73019, USA

**Keywords:** atherosclerosis, biomarkers, ischemic heart disease, lipids, multi-omics, proteome

## Abstract

Premature coronary artery disease (PCAD) is a growing public health concern, especially in South Asia, where traditional risk factors fail to fully explain the increasing incidence of early-onset myocardial infarction. To explore its molecular underpinnings, we conducted a pilot study analyzing plasma proteins and lipids to identify potential biomarkers and dysregulated pathways associated with PCAD. Label-free quantitative proteomics revealed distinct molecular signatures separating PCAD patients from age- and sex-matched healthy controls. Key alterations included upregulation of GALE, immunoglobulin genes, and KIF20B, suggesting enhanced inflammatory responses and proliferative activity associated with post-myocardial infarction cellular repair. Similarly, down regulations of various proteins linked to multiple functions, such as myocardial infarction, hemoglobinopathy, complement and coagulation cascade, and fatty acid and lipoprotein transport in hepatocytes, were observed. Untargeted lipidomics further revealed significant elevations in several phosphatidylcholine species (PC 42:5, PC 40:3, and PC 42:7), highlighting disruption of highly unsaturated phospholipid metabolism. Overall, these findings indicate that PCAD is a multifactorial disorder involving metabolic, immune, and vascular dysfunction beyond conventional lipid abnormalities, underscoring the need for larger cohort studies to validate these biomarkers and uncover novel therapeutic targets.

## 1. Introduction

Coronary artery disease (CAD) remains the leading cause of mortality worldwide, accounting for approximately 8.9 million deaths in 2015 according to the Global Burden of Disease Study [1]. Of increasing concern is the rising incidence of premature coronary artery disease (PCAD), defined as myocardial infarction (MI) occurring before 45 years in men and 55 years in women, with an even more severe subset affecting individuals under 40 years. In one large cohort, 21% of MI patients were younger than 40 years, 79.8% of whom were men [2]. Similarly, the incidence of acute MI increases sharply from 2.1 per 100,000 person-years among those aged 20–29 to 16.9 among those aged 30–39 [3]. Southeast Asia is experiencing a particularly rapid expansion of this burden. In Pakistan, the world’s fifth most populous nation, persistent public health challenges, such as resource limitations, regional instability, and inadequate infrastructure, compound the impact of non-communicable diseases. Although infectious diseases remain prevalent, CAD has become the leading cause of death. In 2019, the country reported an age-standardized cardiovascular disease incidence rate of 918.18 per 100,000 compared with a global rate of 684.33, and a mortality rate of 357.88 per 100,000 compared with the global rate of 239.85 [4].

Risk factor distributions vary substantially across ethnicities. In Black populations, dyslipidemia, diabetes, and smoking are major drivers, while in Hispanics, male sex, dyslipidemia, and family history predominate. Among Whites, smoking remains the strongest predictor [5]. Interestingly, no single independent clinical predictor has been identified in Asian Indians. Western population studies reveal the highest PCAD prevalence among American Indians and Whites (~50%), followed by African Americans (30%) and Hispanics (20%) [6]. In South Asian populations, however, family history and genetic predisposition appear dominant [7,8]. Despite apparently “normal” lipid profiles, they often display aggressive atherosclerosis and higher cardiovascular mortality [9]. Although hypertension, diabetes, and smoking are more prevalent and often undertreated in South Asians, these factors alone do not account for the disproportionate risk. The INTERHEART study identified higher odds of MI associated with elevated ApoB100/ApoA-I ratios, psychosocial stress, and inadequate control of modifiable risk factors [10].

Traditional epidemiological approaches, such as polygenic risk scores, have revealed inherited predispositions to atherosclerosis independent of conventional risk markers [11]. Similarly, imaging modalities, such as coronary artery calcium (CAC) scoring and coronary CT angiography, are increasingly enhanced by artificial intelligence to refine phenotypic risk assessment [12]. However, these methods capture only part of the disease complexity, providing information on risk or burden rather than underlying mechanisms. Emerging evidence indicates that subtle inflammatory and metabolic disturbances, often clinically silent, play crucial roles in the initiation of CAD [13,14].

Identifying circulating biomarkers offers complementary molecular insight, though only a few, such as C-reactive protein (CRP), have been widely adopted clinically [15]. Modern multi-omics approaches, particularly proteomics and lipidomics, enable direct investigation of functional molecular alterations in protein and lipid profiles underlying atherosclerosis, substantially enhancing risk prediction beyond conventional factors [16,17]. For example, recent proteomic studies have identified proteins such as GDF15 and MMP12 as strong predictors of incident CAD across diverse age and ethnic groups [18].

In the present study, we conducted integrated LC-MS/MS-based proteomic and lipidomic analyses to compare PCAD patients with matched controls, aiming to better understand the molecular basis of early disease onset and to improve individualized risk stratification. While genetic variants offer important insights, proteomic profiling and untargeted lipidomic analyses provide a more direct assessment of their functional consequences, including differential expression, structural alterations, and pathway dysregulation at the protein level. This multi-omics approach helps bridge the gap between genetic predisposition and phenotypic expression, offering deeper insight into the molecular drivers of CAD in high-risk populations and potentially guiding the development of population-specific diagnostics and therapeutic strategies.

## 2. Results

### 2.1. Characteristics of PCAD Patients

Healthy control volunteers were all males with an average age of 41.3 ± 0.6 years. One participant reported a prior history of dyslipidemia, and one had Type 2 diabetes mellitus. None had prior hypertension, peripheral arterial disease, MI, or PCI, and none were taking medications at the time of sampling. The mean age of PCAD cases was 41.7 ± 0.6 years. All patients had experienced ST-elevation myocardial infarction (STEMI). One had a history of hypertension, and one had controlled Type 2 diabetes mellitus (A1C < 6.5%). None had prior dyslipidemia, myocardial infarction, percutaneous coronary intervention (PCI), or other coronary artery disease (CAD) equivalents. Lipid profiling at index hospitalization revealed only one patient meeting hyperlipidemia criteria. All patients were prescribed appropriate post-MI therapy, including aspirin, P2Y12 antagonists, beta-blockers, and statins, with rosuvastatin being most common.

### 2.2. Plasma Proteomic Landscape of PCAD Patients

**Figure 1:**
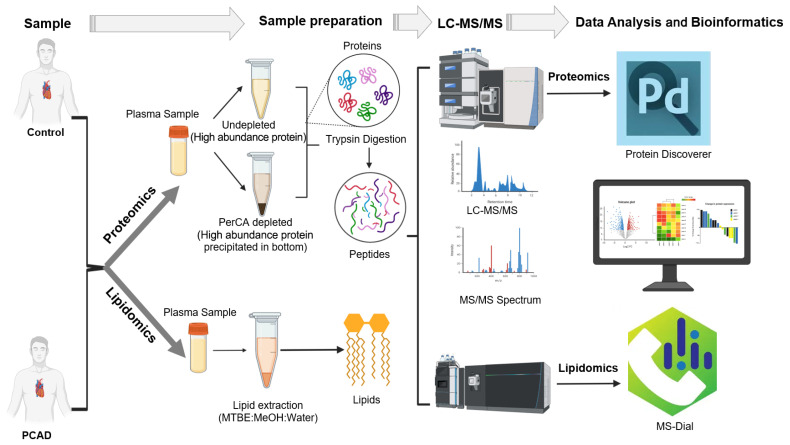
Experimental workflow of integrated plasma proteomics and lipidomics analysis comparing PCAD patients and control samples.

To examine profiles of both abundant and low-abundance, low-molecular-weight plasma proteins, this study employed two plasma preparation workflows: undepleted and PerCA-depleted plasma, followed by label-free quantitative proteomic analysis (Figure 1).

**Figure 2:**
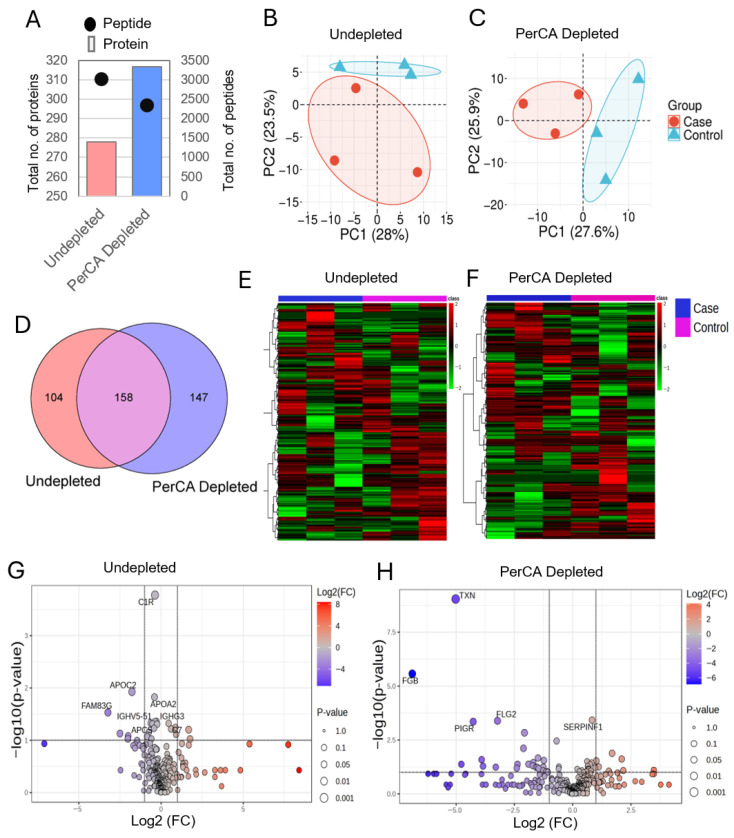
Comparative proteomic profiling of PCAD plasma samples. (**A**) Bar diagram shows the total number of proteins/peptides identified and quantified. (**B**,**C**) Principal component analysis (PCA) of normalized protein abundance values showing distinct grouping patterns that illustrate clear separation between PCAD (red) and control (pale blue) samples in both methods. (**D**) Proportional Venn diagram analysis shows the unique and overlapped genes identified by each method. (**E**,**F**) Hierarchical clustering heatmap of normalized protein intensities showing differential expression profiles across all samples. Data were normalized by median, log_2_ transformed, and auto-scaled; clustering was based on Euclidean distance and Ward’s linkage method. Red = upregulated, green = downregulated relative to the mean. (**G**,**H**) Volcano plot showing proteins with ≥1.5-fold change and *p* < 0.05. Red and blue points indicate up- and downregulated proteins, respectively, in PCAD cases.

A total of 278 and 317 protein groups were successfully identified and quantified from undepleted and PerCA-depleted plasma samples, respectively (Figure 2A, Tables S1 and S2). Principal component analysis (PCA) of overall protein abundance revealed clear global proteomic differences between control and case samples for both extraction methods (Figure 2B,C). A qualitative comparison of the unique genes identified in undepleted versus PerCA-depleted plasma revealed substantial differences in the protein profiles between these two approaches (Figure 2D).

Gene enrichment analysis of these unique protein sets showed that each method captures proteins involved in distinct biological pathways (Figure S1), underscoring the critical role of depletion strategies in enhancing the identification and quantification of low-abundance proteins. The accuracy of the label-free quantitative analysis was further validated using the internal control protein (yeast enolase), which achieved approximately 84–90% sequence coverage in both methods. Peak area-based quantification (LFQ) showed no significant variation, indicating that the sample processing and downstream analysis steps, including plasma protein depletion, trypsin digestion, desalting, peptide enrichment, and LC-MS/MS analysis were consistent (Figure S2). Heat map analysis of the total number of unique proteins identified by each method further highlights differences in protein abundance between control and PCAD case samples (Figure 2E,F). Although no distinct clusters exhibited a consistent pattern of upregulation or downregulation, quantitative analysis demonstrated workflow-specific alterations in protein abundance between PCAD cases and controls. Several proteins identified within the undepleted and PerCA-depleted workflows showed significant changes exceeding a log_2_ fold change threshold of 1.5 (Figure S3). Additionally, volcano plot analysis identified nine significantly altered proteins in the undepleted method and 30 in the PerCA-depleted method, each demonstrating at least a 1.5-fold change and a *p*-value < 0.05 between PCAD case and control samples (Figure 2G,H).

### 2.3. Functional Annotation and Pathway Enrichment Analysis of Differentially Altered Proteins

**Figure 3:**
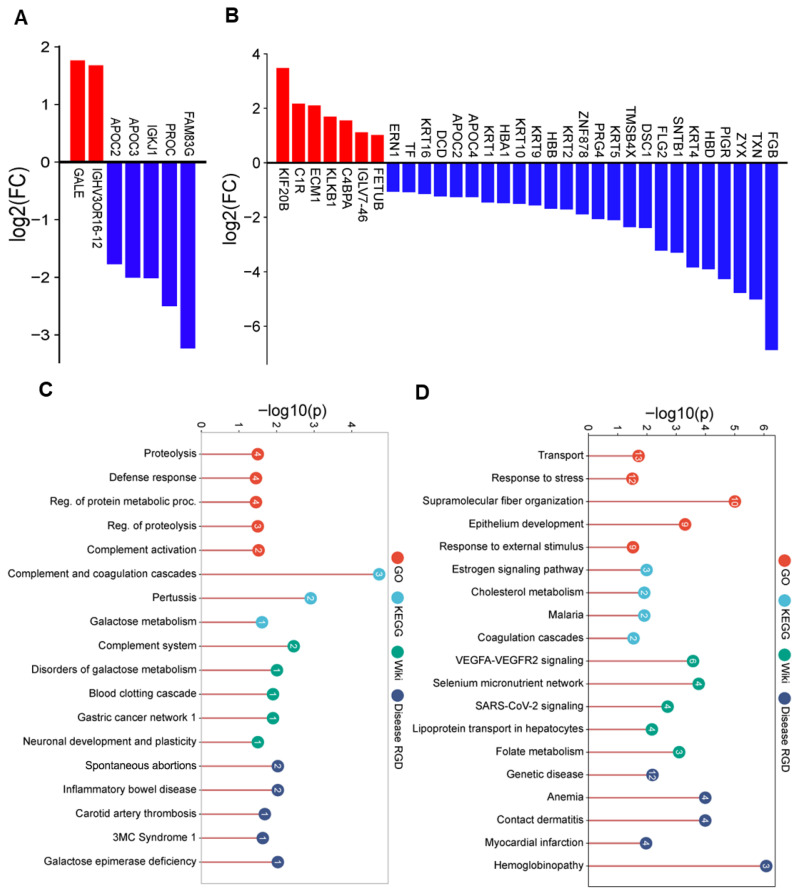
Differentially expressed serum proteins between PCAD cases and controls. (**A**,**B**) Bar diagrams showing differentially expressed proteins between PCAD cases and controls identified using undepleted (**left**) and PerCA-depleted (**right**) serum proteomic workflows. Proteins were selected based on ≥1.5-fold change and *p* < 0.05. Red and blue bars indicate proteins with higher and lower abundance, respectively, in PCAD cases relative to controls. (**C**,**D**) Integrated pathway enrichment analysis of differentially expressed proteins identified from the undepleted (**C**) and PerCA-depleted (**D**) datasets. Enrichment results from KEGG (Blue), Gene Ontology (biological process) (Red), WikiPathways (Green), and Disease RGD (Dark Teal) databases were combined into a unified visualization generated using SRplot based on ShinyGO analysis. Each dot represents an enriched pathway or functional term, the *x*-axis indicates statistical significance (−log10 *p*-value), dot color denotes the corresponding enrichment database category, and the number displayed within each dot represents the number of genes associated with the enriched pathway.

Applying a ≥1.5-fold change and *p* < 0.05 cutoff, we identified 39 proteins that were significantly altered in PCAD samples compared to controls across both methods (Figure 3A,B). Of these, 7 and 32 proteins were differentially abundant in undepleted and PerCA-depleted samples, respectively. Among the significantly altered proteins, nine were increased in PCAD samples: GALE and IGHV3OR16-12 in the undepleted group, and KIF20B, C1R, ECM1, KLKB1, C4BPA, IGLV7-46, and FETUB in the PerCA-depleted samples. In contrast, several predominant plasma proteins, including APOC2, APOC3, APOC4, FGB, IGKJ1, vitamin K-dependent protein C (PROC), hemoglobin subunits (delta (HBD), beta (HBB), and alpha (HBA1)), TXN and ZYX, were decreased in PCAD samples relative to controls.

Functional enrichment analyses, including Gene Ontology (GO), Kyoto Encyclopedia of Genes and Genomes (KEGG), WikiPathways, and Disease RGD databases, of the significantly altered proteins revealed distinct yet complementary enrichment patterns (Figure 3C,D). Proteins with increased abundance in PCAD were predominantly associated with proteolysis (ECM1, KLKB1, C1R, FETUB), complement and coagulation cascades (KLKB1, C1R, C4BPA), and galactose metabolism (GALE), suggesting enhanced coagulation activity and thrombo-inflammatory processes in PCAD cases (Figure 3C).

In contrast, downregulated proteins were mainly enriched in pathways related to lipid transport, extracellular matrix organization, and platelet function, indicating potential disruptions in vascular integrity and lipid metabolism (Figure 3D). Disease RGD pathway analysis further showed that proteins linked to myocardial infarction (APOC3, TXN, FGB, PROC) and hemoglobinopathies (HBB, HBA1, HBD) were significantly decreased in PCAD samples (Figure 3D).

Overall, the PerCA-depleted dataset identified a broader range of differentially expressed proteins, including several low-abundance proteins not detected in the undepleted workflow. Many of these proteins are associated with cardiovascular and metabolic pathways, highlighting that PerCA depletion enhances the detection of biologically relevant low-abundance plasma proteins linked to disease.

### 2.4. Untargeted Comparative Lipidomic Profiling of PCAD Plasma Samples

**Figure 4:**
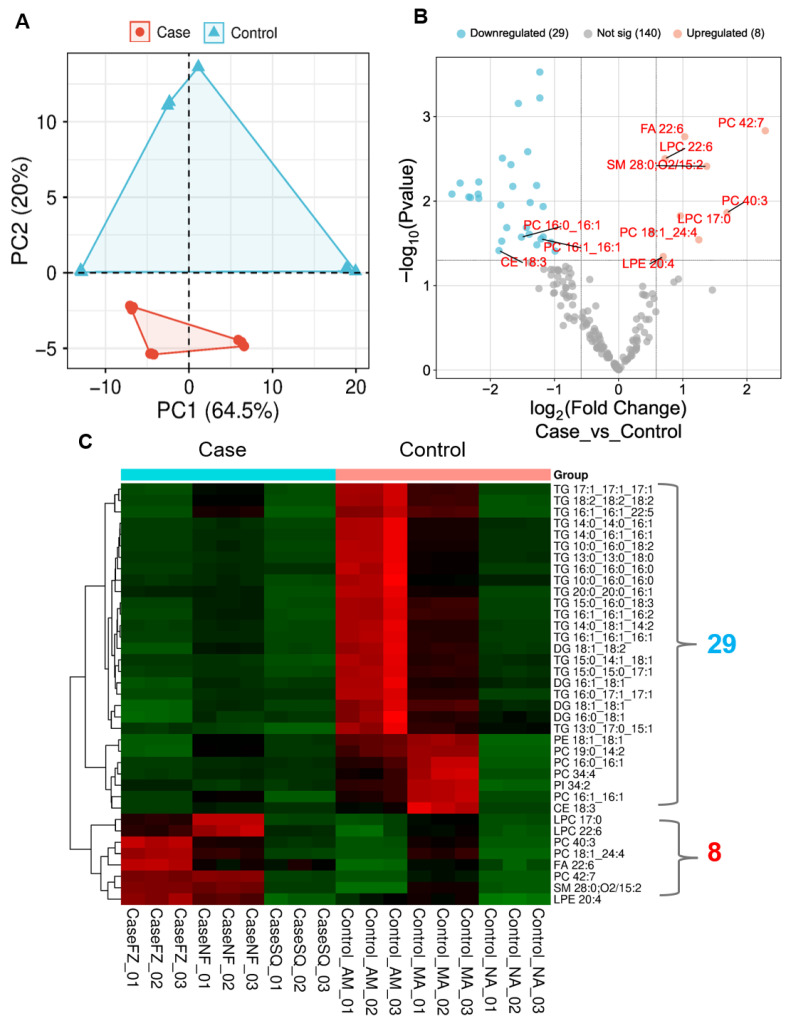
Comparation of plasma lipidomic profiles between PCAD patients and control group. (**A**) PCA of PCAD case (red) and control (green) samples. (**B**) Volcano plot represents significantly increased (red) and decreased (pale) lipids. Gray dots are not statistically significant. (**C**) Heat map analysis represents abundance profile of normalized lipid intensities showing differential expression profiles across all samples. Data were normalized by median, log_2_ transformed, and auto-scaled; clustering was based on Euclidean distance and Ward’s linkage method. Red = upregulated, green = downregulated relative to the mean.

In this study, we successfully identified and quantified a total of 177 lipids (manually checked, high confidence) from PCAD plasma samples (Table S3). Principal component analysis (PCA) revealed a clear separation between PCAD cases and controls; however, the control samples exhibited greater dispersion, indicating higher variability in their plasma lipid profiles (Figure 4A). We next identified lipids that were significantly altered in PCAD samples compared to controls (*p* < 0.05 and fold change < −1.5 or >1.5). Volcano plot analysis further highlighted these differentially abundant lipids (Figure 4B). Heatmap analysis illustrated the abundance patterns of the 65 significantly altered lipids, of which 29 were decreased and 8 were increased in PCAD plasma samples (Figure 4C). We further examined the structural features of triglycerides (TGs) to assess whether the observed changes were associated with specific chain lengths or degrees of unsaturation. Interestingly, the subset of downregulated TGs shows a distinct structural pattern. These TGs are predominantly composed of shorter chains and more saturated or moderately unsaturated fatty acyl chains (e.g., TG 16:0_16:0_16:0, TG 14:0_14:0_16:1, TG 15:0_15:0_17:1). In addition, several TGs containing odd-chain fatty acids (e.g., C13, C15, C17) and lower degrees of unsaturation were also decreased. Only a limited number of highly polyunsaturated TGs (e.g., TG 16:1_16:1_22:5 and TG 18:2_18:2_18:2) were observed in the downregulated group (Figure 4C).

## 3. Discussion

This pilot study demonstrates the feasibility of a multi-omics platform, integrating proteomics and lipidomics for the analysis of PCAD patients, and highlights distinct molecular alterations associated with early vascular dysfunction. Proteomic profiling was performed on both undepleted plasma and PerCA-depleted samples to capture complementary layers of circulating proteins, including low-abundance species. Overall, fewer proteins exhibited increased abundances in the PCAD group (Figure 3). In undepleted plasma, only two proteins, GALE and IGHV3-OR16-12, were significantly elevated in PCAD compared to controls. Glycosylation is critical for normal megakaryopoiesis and platelet production, and increased glycosylation activity may reflect a prothrombotic state [19,20]. GALE, a key enzyme involved in galactose metabolism and protein and lipid glycosylation, was increased in PCAD, supporting the concept that glycoprotein remodeling contributes to early atherogenesis through its effects on thrombosis, inflammation, immune activation, and lipoprotein pathogenicity [19]. Similarly, the increased abundance of IGHV3-OR16-12 in PCAD suggests enhanced humoral immune activity and expansion of the B-cell repertoire in PCAD patients. This finding is consistent with antigen-driven immune responses observed in atherosclerosis, potentially targeting oxidized lipoproteins, modified ApoB, or endothelial neo-epitopes [21,22]. The attenuation of this signal following PerCA depletion further supports the presence of an immunoglobulin-dependent circulating immune profile in PCAD.

Proteins upregulated in the PerCA-depleted plasma fraction predominantly reflect inflammatory, immune, and reparative processes that may be amplified in the post-myocardial infarction (MI) setting in PCAD (Figure 3), although some signals may also point to mechanisms underlying early-onset atherosclerosis. Upregulation of KIF20B likely indicates increased proliferative activity associated with post-MI cellular repair and remodeling rather than a primary predisposing mechanism [23]. In contrast, increased C1R abundance may reflect a dual process, involving activation of the classical complement pathway implicated in early atherogenesis [24,25], along with a superimposed post-MI inflammatory or reparative response [26]. Similarly, increased levels of ECM1, KLKB1, and C4BPA are consistent with vascular injury-associated repair, remodeling, and regulation of inflammatory and complement pathways [27,28,29]. Notably, FETUB, a liver-derived hepatokine, emerged as a particularly compelling candidate due to its reported associations with systemic inflammation, insulin resistance, diabetes, metabolic syndrome, and atherosclerosis [30,31]. Although contributions from myocardial injury-related stress responses cannot be excluded, the biological plausibility of FETUB in early atherogenesis warrants further investigation in larger, well-characterized cohorts.

In contrast, many proteins showed decreased abundance in the PCAD group within the PerCA-depleted samples, reflecting dysregulation of lipid metabolism, immune activation, coagulation, and intracellular signaling. APOC2, a key activator of lipoprotein lipase, was reduced, suggesting impaired triglyceride-rich lipoprotein processing and remnant clearance, mechanisms relevant to early-onset coronary artery disease [32,33]. Interestingly, APOC3, an inhibitor of lipoprotein lipase, also decreased, likely due to lipid-lowering therapies among PCAD cases [34,35]. Immune-related changes included increased IGKJ1, indicating heightened B-cell and plasma-cell activity, consistent with humoral immune contributions to atherosclerosis [21,22]. Reduced PROC, a vitamin K-dependent anticoagulant, suggests compromised anticoagulant and endothelial protective mechanisms, favoring a prothrombotic and pro-inflammatory vascular environment [36,37]. Downregulation of FAM83G (PAWS1), involved in Wnt/β-catenin and cytoskeletal signaling, points to impaired intracellular stress responses, potentially affecting vascular smooth muscle and endothelial function [38]. Lower fibrinogen beta chain (FGB) likely reflects consumption in activated coagulation, as expected post-MI [39]. Other downregulated proteins with mechanistic relevance include TXN, a key antioxidant, and ZYX, a focal-adhesion protein, suggesting oxidative stress, impaired mechanotransduction, and early atherogenesis [40,41,42].

Several structural and barrier-associated proteins, including PIGR, SNTB1, and FLG2, were reduced, potentially increasing endothelial vulnerability and promoting early atherosclerosis [43,44]. Reduced TMSB4X and PRG4, involved in cytoskeletal repair and anti-inflammatory responses, along with decreased ERN1, a stress-response regulator, indicate compromised endothelial repair and remodeling [45,46,47,48]. Some proteins likely reflect systemic stress rather than atherosclerosis, including hemoglobin subunits (HBD, HBB, HBA1) and DSC1, ZNF878, DCD, and TF [49,50,51,52]. Finally, APOC4, involved in triglyceride-rich lipoprotein handling, decreased in abundance; although its role in atherosclerosis is not fully established, it warrants further study [51].

Similar to proteomic findings, only a subset of lipids, primarily phospholipid species, were significantly elevated in the PCAD group compared with healthy controls, indicating altered membrane lipid remodeling (Figure 4). Several phosphatidylcholine (PC) species, including PC 18:1_24:4, PC 40:3, and PC 42:7, were increased, suggesting dysregulation of highly unsaturated phospholipid metabolism. PCs are major structural components of circulating lipoproteins and cellular membranes and play key roles in lipid transport and membrane integrity [53]. Prior studies linked altered PC profiles with oxidative stress, lipoprotein remodeling, and endothelial dysfunction in atherosclerosis. In addition, increased PC levels have been associated with greater epicardial adipose tissue volume, a precursor of atherosclerosis [54]. Large cohort studies further demonstrate that specific PC species are associated with cardiovascular outcomes, with PCs containing saturated or monounsaturated fatty acids positively associated with cardiovascular risk, while PCs enriched with polyunsaturated fatty acids show inverse associations [55]. Increased sphingomyelin (SM 43:2;O2) in the PCAD group also suggests sphingolipid remodeling and association with cerebrovascular atherosclerosis, cardiometabolic syndromes, and coronary artery disease [56,57].

Furthermore, increased abundances of several phospholipids, including docosahexaenoic acid (DHA, FA 22:6) and lysophospholipids such as LPC 17:0, LPC 22:6, and LPE 20:4, suggest increased phospholipid turnover and inflammatory lipid signaling. DHA is generally associated with reduced atherosclerotic risk, although its elevation in PCAD may reflect compensatory anti-inflammatory signaling in response to vascular inflammation and lipid remodeling [58]. Lysophosphatidylcholines are bioactive lipids generated through phosphatidylcholine hydrolysis and are abundant components of oxidized LDL and atherosclerotic plaques [59]. Increased LPC levels may therefore reflect enhanced lipoprotein oxidation and inflammatory signaling associated with early atherosclerosis [60]. Similarly, increased LPE 20:4 may indicate phospholipase-mediated membrane phospholipid turnover during vascular inflammation and ischemic injury, representing metabolic signatures of inflammatory lipid remodeling rather than direct pathogenic mediators of atherosclerosis [61]. In contrast, several neutral lipids, including triglycerides and diglycerides, were significantly decreased in the PCAD group, possibly reflecting increased lipolysis, metabolic utilization, or lipid remodeling following myocardial injury. Reduced levels of CE 18:3 may also reflect cholesterol utilization during plaque formation, while differential changes in some PC species (e.g., decreased PC 32:1 and PC 34:2) suggest complex lipid remodeling processes that warrant further investigation in larger-scale studies.

## 4. Materials and Methods

### 4.1. Study Design

A case (PCAD patients) control study design was employed to investigate proteomic alterations associated with PCAD patients. Plasma samples were collected from patients with clinically diagnosed PCAD (*n* = 3) and from age-matched healthy controls (*n* = 3), following standardized pre-analytical protocols. All participants provided written informed consent in accordance with institutional ethical guidelines. The study protocol was approved by the Institutional Review Board of Armed Forces Institute of Cardiology, Rawalpindi, Pakistan (Reg#:S/189/2024; IERB#:13/24).

### 4.2. Study Participants and Sample Collection

Eligible cases were men aged <43 years with a recent diagnosis of MI confirmed by clinical history, ECG findings, and troponin elevation. Controls were free of clinical CAD and matched to PCAD patients based on sex and an age of 41 years (+/−1 year). Exclusion criteria included known inflammatory or autoimmune disease, malignancy, or chronic kidney disease stage 3 or above. Demographic data, cardiovascular risk factors, and medication history were recorded. Plasma samples from each patient and control were collected according to clinically approved protocol. Detailed participant information is provided in Table S4.

### 4.3. Protein Extraction and Sample Processing for Proteomic Analysis

Two different sample treatment methods, hereafter referred to as undepleted and perchloric acid (PerCA), were used to isolate plasma proteins from PCAD patient samples. Total protein concentration was measured using a NanoDrop™ One microvolume UV–Vis spectrophotometer (Thermo Fisher Scientific, Madison, WI, USA). Detailed protein extraction protocols have been described in our previous reports [62,63]. A total of 100 µg of protein from each plasma sample was subjected to in-solution proteolytic digestion [62,63]. Prior to proteolysis, 1 µg of *Saccharomyces cerevisiae* enolase (Sigma-Aldrich, St. Louis, MO, USA) was added as an internal digestion control. Samples were reduced with 10 mM dithiothreitol (DTT) at 37 °C for 1 h and alkylated with 20 mM iodoacetamide (IAA) in the dark for 30 min. The urea concentration was then diluted to <1 M before overnight digestion with sequencing-grade Trypsin/Lys-C (Promega, Madison, WI, USA) at 37 °C using an enzyme-to-substrate ratio of 1:50. Resulting peptides were desalted using Waters C18 Sep-Pak Plus cartridges (cat. no. WAT023590, Waters, Milford, MA, USA), vacuum-dried using a SpeedVac concentrator (Savant SVC 100H, Thermo Fisher Scientific, Waltham, MA, USA), and reconstituted in 100 µL of 0.1% formic acid (FA). An aliquot corresponding to 2 µg of tryptic digested peptides was injected for LC-MS/MS analysis.

### 4.4. LC-MS/MS Analysis for Proteomic Analysis

For proteomic analysis, the LC-MS/MS platforms were used as described previously [62,63,64], using a Dionex UltiMate 3000 UHPLC system (Thermo Fisher Scientific, Germering, Germany) coupled to a Q Exactive HF-X mass spectrometer (Thermo Fisher Scientific, Bremen, Germany). Briefly, peptides were separated on a Thermo EasySpray C18 analytical column (3 µm, 75 µm × 15 cm, ES900, Thermo Fisher Scientific, Germering, Germany). The total run time was 60 min, employing a linear gradient of 5% to 35% acetonitrile (ACN) over 30 min at a flow rate of 350 nL/min, followed by a rapid ramp to 95% ACN for column washing and a 10 min re-equilibration at 5% ACN. Electrospray ionization was conducted in positive mode at a spray voltage of 2.1 kV.

### 4.5. Data Analysis for Bottom-Up Proteomics

Acquired MS RAW files were analyzed using the Sequest HT algorithm in Proteome Discoverer v2.4 (Thermo Fisher Scientific, San Jose, CA, USA), following validated analytical pipelines previously described [62,63,64]. Spectra were searched against the UniProt-reviewed *Homo sapiens* database (TaxID: UP000005640) with the following parameters: trypsin enzyme specificity, up to 2 missed cleavages allowed, precursor-ion mass tolerance of 10 ppm, and fragment-ion mass tolerance of 0.02 Da. Dynamic modification of methionine oxidation (+15.9949 Da) and static carbamidomethylation (+57.0215 Da) on cysteine were specified. Peptide and protein identifications were filtered to a 1% false discovery rate (FDR). Label-free quantitation employed the Minora feature detection and alignment algorithm integrated in Proteome Discoverer. Proteins exhibiting a ≥1.5-fold change in abundance and *p* < 0.05 were considered significantly altered.

### 4.6. Lipid Extraction from Plasma Samples

Two-phase extraction was performed following a modified Matyash protocol [65]. All steps were conducted on ice unless noted otherwise. Briefly, 40 µL of plasma sample was mixed with 225 µL of cold methanol (−20 °C) and vortexed for 30 s. A total of 750 µL of MTBE (methyl tert-butyl ether) was added, followed by 2 min of vortexing. A further 188 µL of LC-MS grade water (Optima, Thermo Fisher Scientific, Waltham, MA, USA) was then added, and the mixture was vortexed for 1 min. Phase separation was achieved by centrifugation at 16,000× *g* for 2 min at 15 °C. The organic (upper) phase (300 µL) was collected and dried under SpeedVac (Thermo Fisher Scientific, Waltham, MA, USA) for 2 h at 45 °C. For LC-MS analysis, the sample was resuspended in 100 µL of methanol:MTBE (1:1, *v*/*v*).

### 4.7. LC-MS/MS Analysis for Lipidomics

LC-MS/MS experiments were performed using a Vanquish UHPLC system (Thermo Fisher Scientific, Waltham, MA, USA) coupled to an Orbitrap Eclipse Tribrid mass spectrometer (Thermo Fisher Scientific, Bremen, Germany). A total of 4 µL of sample was injected onto a Kinetex C18 column (1.7 μm, 100 Å, 100 × 2.1 mm; Phenomenex, Torrance, CA, USA; P/N 00D-4475-AN). The autosampler was maintained at 15 °C, and the column oven was at 45 °C (forced-air mode). The flow rate was 0.30 mL min^−1^. Mobile phases were as follows: solvent A, 60% acetonitrile (ACN) and 40% water containing 10 mM ammonium formate and 0.1% formic acid; solvent B, 90% isopropanol (IPA) and 10% ACN containing 10 mM ammonium formate and 0.1% formic acid. The gradient was 30% B (0–1.0 min), 43% B (5.0 min), 50% B (5.5 min), 70% B (14.0 min), 99% B (26.0–29.0 min), returned to 30% B in 0.5 min, and re-equilibrated at 30% B for 5 min. Heated electrospray ionization (H-ESI) was operated in positive and negative ion modes with spray voltages of +3.5 kV and −3.0 kV, respectively. Source settings were sheath gas, 50 (arbitrary units); auxiliary gas, 10 (arbitrary units); sweep gas, 2 (arbitrary units); ion-transfer tube temperature, 320 °C; and vaporizer temperature, 300 °C. The RF lens was set to 40%. Full MS (MS^1^) scans were acquired in the Orbitrap at a resolving power of 240,000 (at *m*/*z* 200) over *m*/*z* 200–1600 with an AGC target of 1.0 × 10^6^ (250%) and a maximum injection time of 50 ms. A cycle time of 1.2 s and an intensity threshold of 5 × 10^3^ were applied. MS/MS (MS^2^) spectra were acquired in the ion trap using quadrupole isolation (isolation window, 0.7 *m*/*z*) and higher-energy collisional dissociation (HCD) with stepped normalized collision energies of 22%, 27%, and 32%. The ion-trap scan rate was set to rapid with dynamic maximum injection time.

Three pooled QC samples (run at the beginning, middle, and end of the sequence) were run to monitor instrument stability and reproducibility. The middle QC sample was used as a reference for retention time and mass alignment. Additionally, two blank samples (run at the beginning and end of the sequence) were included to identify and remove background signals and potential false-positive features.

### 4.8. Lipidomic Data Processing

Raw LC-MS data files were processed in MS-DIAL (version 5.5.251021) [66] using the LipidBlast spectral library (file: Msp20250929174203_NCDK_conventional_converted_dev). For retention-time alignment, the RT tolerance was set to 0.1 min. The MS^1^ mass tolerance for alignment was 0.005 Da, and the MS/MS mass tolerance was 0.025 Da. The MS/MS spectral similarity threshold for alignment was 0.8 (unitless). All lipid annotations were manually reviewed [66,67], and only high-confidence identifications were retained for downstream analyses. Quantification was performed after filtering out lipids with blank annotations, wrong adduct assignments, poor peak shapes, and inconsistent or low-quality MS/MS spectra. Following manual verification and realignment of peaks in MS-DIAL, lipid peak heights were exported and used for quantitative analysis.

### 4.9. Bioinformatic Analysis

Statistical and visualization analyses were conducted using MetaboAnalyst 6.0 [68]. The following normalization parameters were applied: sample normalization by median, log_2_ transformation, and auto-scaling (mean-centered and divided by the standard deviation of each variable). These parameters were selected to minimize technical variation, stabilize variance across features, and improve comparability (PCA), heatmaps, volcano plots, and box plots. The PCA plot heatmaps and volcano plot were generated using SRplot, a free online software for data visualization and graphing [69]. Figure 1 illustrates the detailed analytical pipeline, ensuring consistent sample handling, efficient protein digestion, and high reproducibility across all LC-MS/MS runs and subsequent data analysis.

## 5. Conclusions

This pilot study demonstrates that a multi-omics approach integrating proteomic and lipidomic analyses can uncover molecular alterations in PCAD patients. We identified dysregulated proteins involved in inflammation, immune activation, lipid metabolism, and endothelial repair, alongside selective changes in phospholipids and lysophospholipids, indicating altered membrane metabolism and inflammatory signaling. These results provide mechanistic insights into early vascular dysfunction and highlight potential molecular candidates for biomarker development. However, the study is limited by its small sample size and the inherent biological variability of human serum proteomes, which may influence sample clustering and downstream interpretation. Therefore, the findings should be considered exploratory and hypothesis-generating. Further research with larger, more diverse cohorts including both male and female patients across different age groups and demographic backgrounds is necessary to validate potential early biomarkers and evaluate their clinical relevance.

## Data Availability

The original contributions presented in this study are included in the article/Supplementary Material. All LC-MS/MS RAW files and results files can be found in the MassIVE database (https://massive.ucsd.edu accessed on 4 June 2026) under the following accessions: MSV000101776 and MSV000101774. Further inquiries can be directed to the corresponding authors.

## Supplementary Materials

The following supporting information can be downloaded at: https://www.mdpi.com/article/10.3390/ijms27135684/s1.

## Abbreviations

CAC	coronary artery calcium
CAD	coronary artery disease
CCA-IMT	common carotid artery intima–media thickness
CRP	high-sensitivity C-reactive protein
CVD	cardiovascular disease
DHA	docosahexaenoic acid
GWAS	genome-wide association studies
HDL	high-density lipoprotein cholesterol
HTN	hypertension
LDL	low-density lipoprotein cholesterol
LPC	lysophosphatidylcholine
MI	Myocardial infarction
NT-proBNP	N-terminal pro–B-type natriuretic peptide
OR	odds ratio
PC	phosphatidylcholine
PCAD	premature coronary artery disease
PCI	percutaneous coronary intervention
SM	sphingomyelin
SNP	single nucleotide polymorphism
STEMI	ST-elevation myocardial infarction
