# Supplementary material for: Exploratory LC-MS/MS-Based Proteomic and Lipidomic Profiling of Plasma Samples from Premature Coronary Artery Disease Patients: A Pilot Study in a South Asian Population
Source: Int J Mol Sci. 2026 Jun 24;27(13):5684. doi: 10.3390/ijms27135684 (PMC13361371; doi:10.3390/ijms27135684)
Supplement: Supplementary file 1 [file ijms-27-05684-s001.zip › ijms-4334755-supplementary.pdf]

Supporting Figures and Table

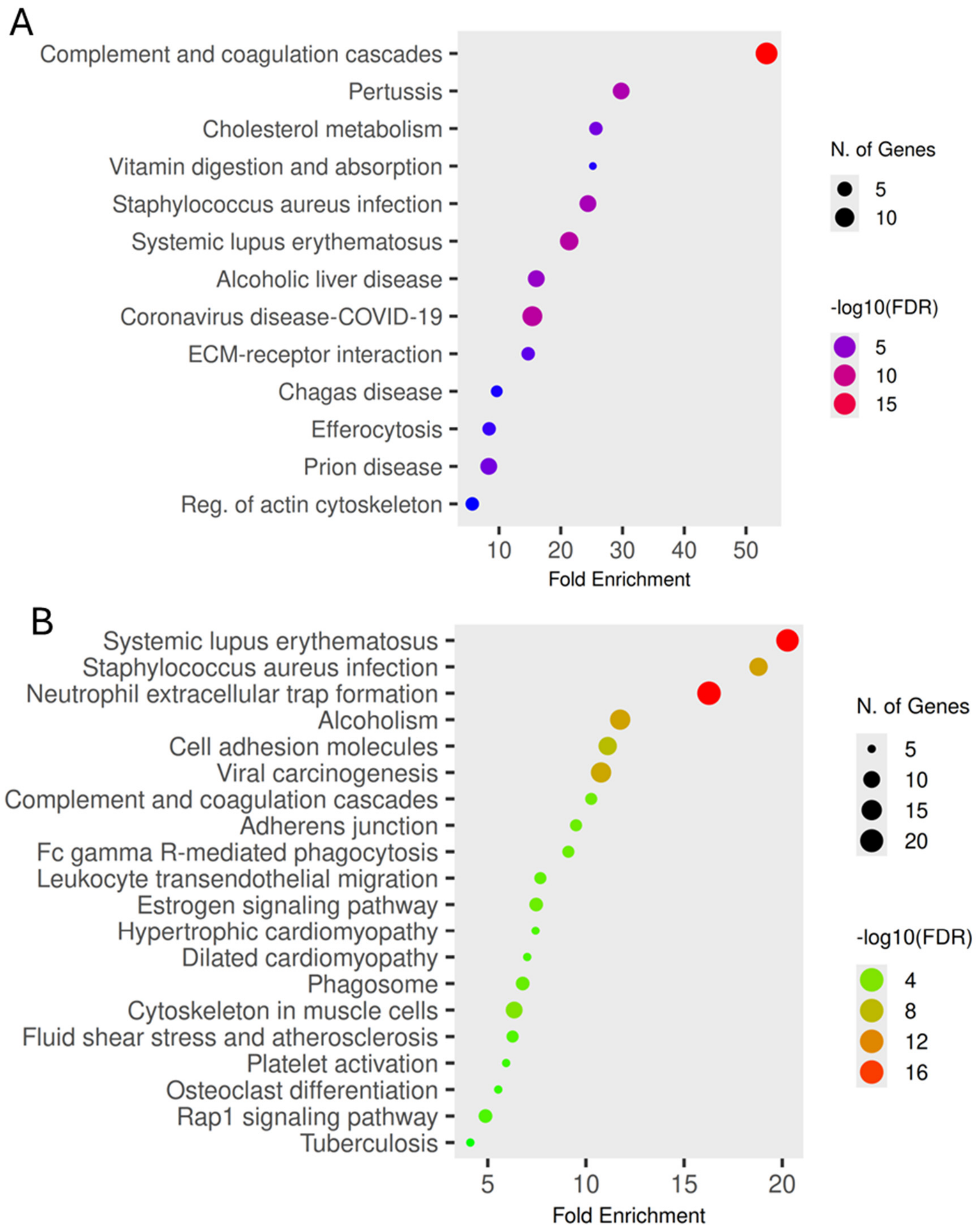

Figure S1. KEGG pathway enrichment analysis of unique sets of plasma proteins identified from undepleted and PerCA depleted PCAD samples. KEGG pathways corresponding to a total of 104 and 147 unique proteins identified in undepleted (A) and

PerCA depleted (B) samples (Figure 2D). Pathways were generated using ShinyGO 0.85.1 an open-source bioinformatics platform.

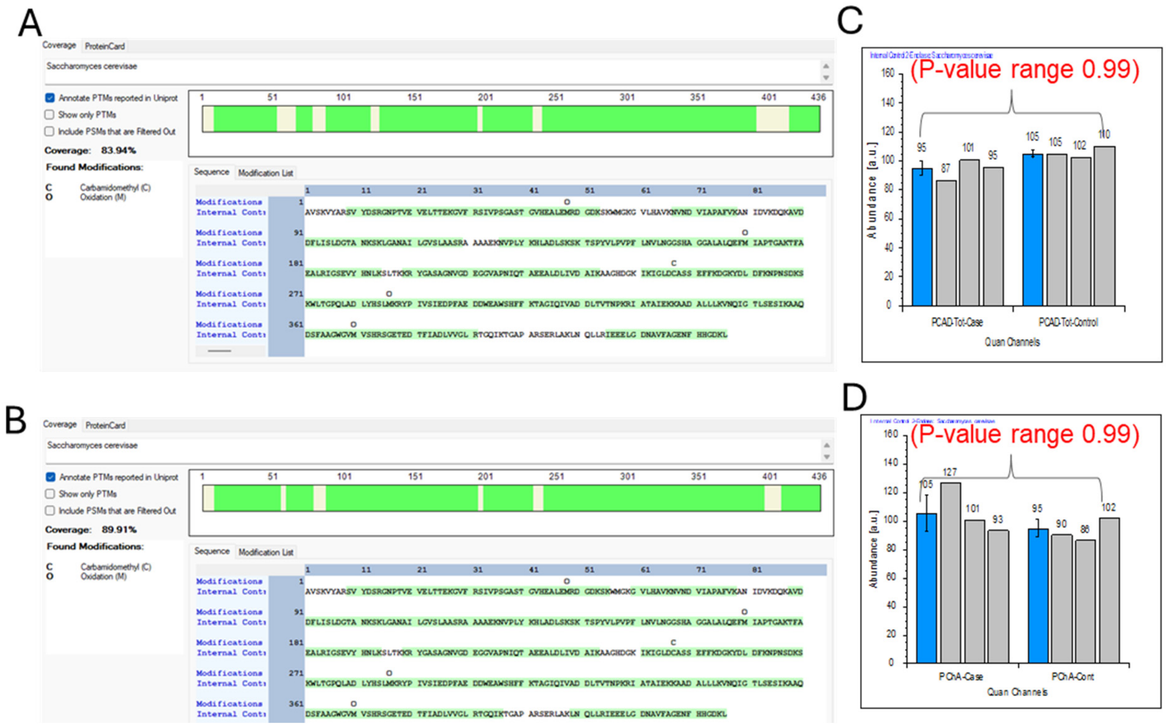

Figure S2. Quality control of the label-free quantitative proteomics of PCAD samples. **A-B** shows the internal control protein sequence and the identified and quantified peptides with green shed from undepleted and PerCA depleted methods. C-D shows the quantitative value (abundance) the internal standard proteins in each sample. Blue bars represent the average value of each group. Gray bar diagrams show the abundance-based quantitative value for internal standard protein in each samples. The p-value shows no significant difference across the samples and conditions. Figures are the direct output of the proteome discoverer (Ver 2.4) software.

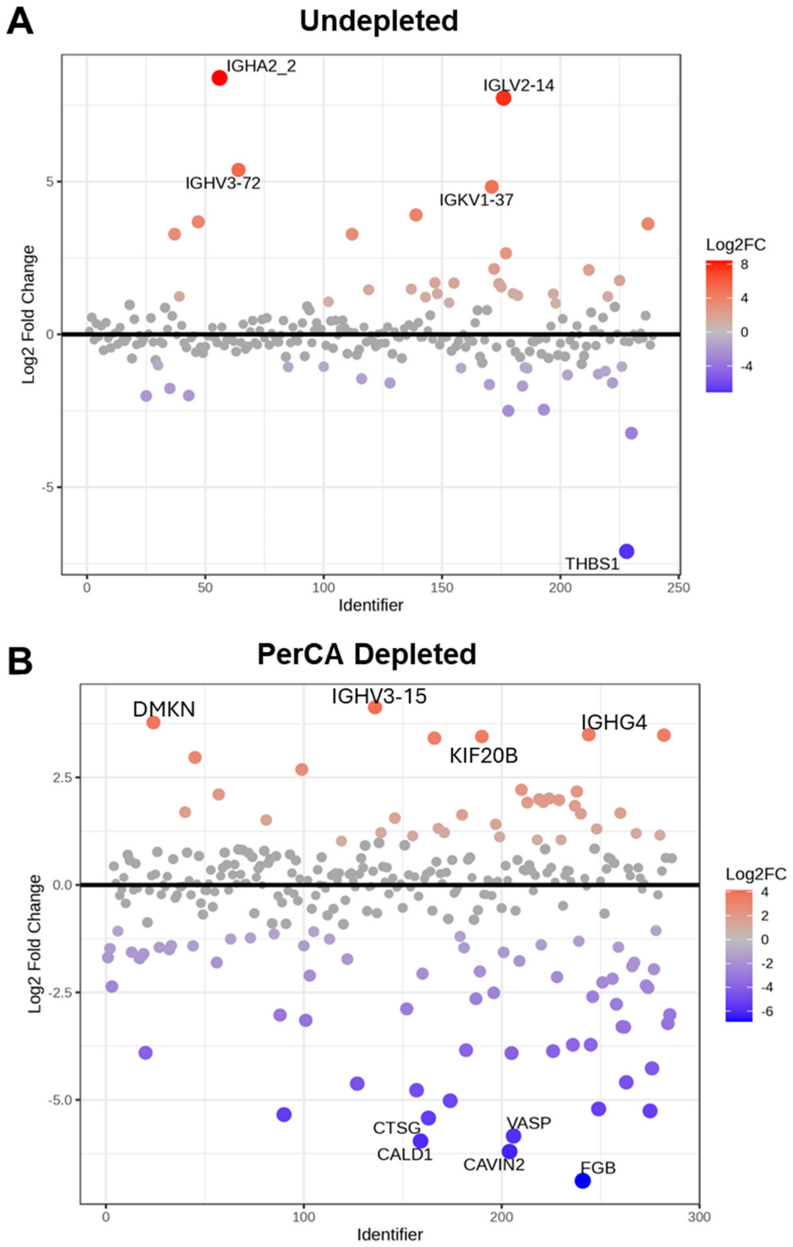

Figure S3. Differential protein expression patterns in PCAD plasma samples in undepleted and PerCA-depleted methods. Log2 fold change distributions of differentially expressed proteins between PCAD cases and controls for undepleted (A) and PerCA-depleted (B) datasets. Each point represents a quantified protein; positive values indicate higher abundance in PCAD cases, and negative values indicate lower

abundance. The horizontal line denotes no change (log2FC = 0). Selected proteins with distinct fold changes are annotated.

Table S4. Baseline demographic and clinical characteristics of PCAD patient and control samples.

Variable	PCAD 1	PCAD 2	PCAD 3	PCAD Overall (n=3)	Control 1	Control 2	Control 3	Control Overall (n=3)
Case ID	904508-08-07	902302-01-12	902302-01-05	—	902303-30-04	902304-10-09	46710-13-09	—
Group	PCAD	PCAD	PCAD	—	Control	Control	Control	—
Age, years	41	42	42	41.7 ± 0.6	42	41	41	41.3 ± 0.6
Hypertension	No	No	Yes	33.3%	No	No	No	0%
Diabetes Mellitus	No	No	Yes	33.3%	No	No	Yes	33.3%
Prior History of Dyslipidemia	No	No	No	0%	No	Yes	No	33.3%
Prior History of CAD	No	No	No	0%	No	No	No	0%
Prior History of PAD	No	No	No	0%	No	No	No	0%
Total Cholesterol, mg/dL	218	86	96	133.3 ± 73.7	134	224	198	185.3 ± 46.2
Triglycerides, mg/dL	139	92	77	102.7 ± 32.4	89	680	138	302.3 ± 330.4
LDL-C, mg/dL	185	42	58	95.0 ± 78.7	100	93	139	110.7 ± 24.9
CRP, mg/L	85.7	1.0	3.1	29.9 ± 48.0	1.0	3.22	7.9	4.0 ± 3.5
HbA1c	U	U	8.8	8.8 (n=1)	5.5	6.3	U	5.9 ± 0.6 (n=2)
Lipoprotein(a), mg/dL	19.2	90.3	8.3	39.3 ± 44.9	4.5	29.3	4.9	12.9 ± 14.2
Blood Glucose, mg/dL	98	U	166	132.0 ± 48.1 (n=2)	U	U	U	Unavailable
GDMT	Yes	Yes	Yes	100%	No	No	No	0%

Abbreviations: CAD = premature coronary artery disease; PAD = Peripheral arterial disease; LDL-C = low-density lipoprotein cholesterol; CRP = C-reactive protein; HbA1c = glycated hemoglobin; U = unavailable; GDMT = Guideline directed medical therapy.
*GDMT = Aspirin, P2Y12 antagonist and statin therapy.
